# RETRACTED: Osman et al. Foliar Spray with Pepsin-and Papain-Whey Protein Hydrolysates Promotes the Productivity of Pea Plants Cultivated in Clay Loam Soil. *Molecules* 2021, *26*, 2805

**DOI:** 10.3390/molecules31111808

**Published:** 2026-05-25

**Authors:** Ali Osman, Abdel-Rahaman M. Merwad, Azza H. Mohamed, Mahmoud Sitohy

**Affiliations:** 1 Biochemistry Department, Faculty of Agriculture, Zagazig University, Zagazig 44511, Egypt; 2 Soil Science Department, Faculty of Agriculture, Zagazig University, Zagazig 44511, Egypt; 3 Agricultural Chemistry Department, Faculty of Agriculture, Mansoura University, Mansoura 35516, Egypt; 4 Citrus Research and Education Center, University of Florida, IFAS, Lake Alfred, FL 33850, USA

The journal retracts the article titled “Foliar Spray with Pepsin-and Papain-Whey Protein Hydrolysates Promotes the Productivity of Pea Plants Cultivated in Clay Loam Soil” [1], cited above.

Following publication, concerns were brought to the attention of the Editorial Office regarding the integrity of an image presented in this publication [1].

In accordance with the journal’s standard procedures, the Editorial Office and Editorial Board conducted an investigation and identified indications of inappropriate image editing contained within Figure 3B. Although the authors cooperated with the investigation, the explanations and supporting materials provided were not deemed sufficient by the Editorial Board to resolve the identified concerns. Consequently, the Editorial Board has lost confidence in the reliability of the findings and decided to retract this publication, as per MDPI’s retraction policy.

This retraction was approved by the Editor-in-Chief of the *Molecules* journal. 

The authors have been informed of this retraction and have indicated their disagreement with this decision.

## References

[1] Osman A., Merwad A.-R.M., Mohamed A.H., Sitohy M. (2021). RETRACTED: Foliar Spray with Pepsin-and Papain-Whey Protein Hydrolysates Promotes the Productivity of Pea Plants Cultivated in Clay Loam Soil. Molecules.

